# Association of the triglyceride-glucose index with carotid intima-media thickness in type 2 diabetes: effect modification by age and albuminuria—a retrospective cross-sectional study

**DOI:** 10.3389/fcvm.2026.1711633

**Published:** 2026-03-04

**Authors:** Yanmei Lin, Kang Du, Jianqing Tian

**Affiliations:** 1 Fujian Medical University Xiamen Humanity Hospital, Xiamen, Fujian, China; 2 Zoe Soft Co. Ltd., Xiamen, Fujian, China

**Keywords:** age, albumin- to-creatinine ratio (ACR), carotid intima-media thickness (IMT), hyperinsulinemia, insulin resistance, TyG index, type 2 diabetes

## Abstract

**Background:**

The Triglyceride-Glucose (TyG) index, a surrogate marker of insulin resistance, is associated with increased cardiovascular risk. However, its relationship with subclinical atherosclerosis in diabetic patients with early-stage kidney injury, characterized by an elevated urinary albumin-to-creatinine ratio (ACR), remains unclear. This study examined the association between carotid intima-media thickness (IMT) and the TyG index, specifically investigating how this association is modified by albuminuria status.

**Methods:**

This cross-sectional study included 507 patients with type 2 diabetes and preserved kidney function who had ACR values of 0–300 mg/g. Participants were stratified into quartiles based on ACR levels. IMT was measured using high-resolution B-mode ultrasonography. The relationship between the TyG index and IMT was evaluated using partial correlation and multivariate linear regression analyses, with emphasis on assessing this relationship across ACR-based strata.

**Result:**

A weak positive correlation was observed between the TyG index and IMT overall (*r* = 0.12, *P* = 0.035), with a significant interaction effect of ACR (*p* = 0.008). Stratification by ACR showed that for patients with ACR ≤ 30 mg/g, each unit increase in the TyG index was associated with a 0.038 mm increase in IMT (95% CI: 0.015–0.061, *p* = 0.002), while for those with ACR > 30 mg/g, the increase was 0.071 mm per unit (95% CI: 0.036–0.106, *p* < 0.001). Furthermore, a graded pattern was observed across ACR quartiles, with the positive correlation between TyG and IMT showing graded strengthening (*β* values increased from 0.022 to 0.078, *p* for trend <0.001;). Finally, the TyG–IMT correlation exhibited age specificity, being significant in patients under 50 years (<40 years: *β* = 0.085, *p* = 0.002; 40–49 years: *β* = 0.051, *p* = 0.015) and diminishing in older age groups.

**Conclusions:**

The association between the TyG index and IMT exhibits stage-specific and population-heterogeneous patterns. A significant, ACR-dependent positive correlation was observed, with the association being pronounced in patients with elevated ACR (>30 mg/g) and in those younger than 50 years. These findings suggest that the pro-atherogenic effect of insulin resistance is more pronounced in diabetic patients with early albuminuria or in younger individuals, underscoring the need for enhanced vascular management to reduce insulin resistance in these high-risk populations.

## 1 Introduction

Carotid artery intima-media thickness (IMT) serves as a crucial subclinical imaging biomarker for atherosclerosis, reflecting the thickness of the arterial far wall. Endothelial dysfunction, an early event in atherogenesis linked to future cardiovascular and cerebrovascular risk, can begin in childhood. Given the pivotal role of subclinical atherosclerosis in predicting cardiovascular events, early identification in high-risk groups is paramount. The rising prevalence of metabolic diseases like type 2 diabetes mellitus (T2DM)—with an estimated 537 million adults affected globally in 2021—underscores this urgency. Patients with T2DM often harbor multiple cardiovascular risk factors, including insulin resistance and dyslipidemia, predisposing them to subclinical atherosclerosis.

The triglyceride-glucose index (TyG index, calculated as ln [fasting triglycerides (mmol/L) × fasting glucose (mmol/L)/2]) has been validated as a reliable surrogate marker for insulin resistance (1), with evidence linking it to arterial structure abnormalities. However, investigations on the TyG-IMT relationship in T2DM patients with preserved kidney function [eGFR ≥ 60 mL/min/1.73 m^2^ and urinary albumin-to-creatinine ratio (ACR) 0–300 μg/mg] remain scarce. This study analyzed the correlation between the TyG index and carotid IMT in 507 hospitalized T2DM patients (2022–2024) and data collection strictly conducted post-approval.

Importantly, we sought to characterize the modifying effect of renal parameters (e.g., albuminuria) on this relationship using a retrospective cross-sectional design (2). The hospitalization setting enabled standardized execution of carotid ultrasound and laboratory assessments, though we acknowledge potential selection bias related to disease severity and treatment intensity. To address this, we quantified exclusion due to creatinine criteria and discussed its directional impact on ACR-metabolic risk distribution. This study provides insights for early cardiovascular risk stratification in T2DM, with findings positioned as hypothesis-generating for future longitudinal and interventional research (3).

## 2 Methods

### 2.1 Study design and participants

This cross-sectional analysis examined the association between the TyG index and IMT in T2DM patients with preserved renal function (eGFR ≥ 60 mL/min/1.73 m^2^) and ACR ≤ 300 mg/g. We retrospectively collected data from patients with T2DM who were hospitalized at Xiamen Humanity Hospital between January 1, 2022, and October 1, 2024. The inpatient setting ensured standardized execution of carotid ultrasound (Philips EPIQ 7C, with inter-operator ICC = 0.92) and laboratory assessments. Inclusion criteria comprised:

Age 20–80 years;
ACR between 0 and 300 mg/g on at least two occasions within the 3 months prior to admission or during hospitalization
Admission serum creatinine levels within the 50–90 μmol/L range (*n* = 42 excluded due to this criterion, representing 7.6% of initial cohort, potentially underestimating ACR-metabolic risk association due to selection of healthier patients).

Key exclusion criteria comprised:

1. eGFR < 60 mL/min/1.73 m^2^;
2. non-diabetic kidney disease;
3. history of acute cardiovascular events within the preceding 6 months;
4. severe liver dysfunction;
5. pregnancy or lactation;
6. history of cervical surgery or carotid revascularization;
7. missing ACR second measurement [*n* = 35, 6.2%, excluded from final analysis].

The study protocol complied with the Declaration of Helsinki and was approved by the Institutional Ethics Committee of Xiamen Humanity Hospital (approval number: HAXM-MEC-20250710-046-01).

### 2.2 Clinical and biochemical measurements

Demographic and clinical data, including age, sex, and smoking status, were collected through interviews and medical record reviews. Body mass index (BMI) was calculated as weight in kilograms divided by height in meters squared (kg/m^2^). After an overnight fast of at least 8 h, venous blood samples were drawn. Fasting plasma glucose (FPG), triglycerides (TG), total cholesterol (TC), low-density lipoprotein cholesterol (LDL-C), serum creatinine, and glycated hemoglobin (HbA1c) were measured using standard automated biochemical analyzers. The urinary ACR was determined from first-morning spot urine samples (4). In accordance with the inclusion criteria, ACR was measured on two occasions within the 3 months prior to admission or during hospitalization. The arithmetic mean of these two measurements was calculated and used for all subsequent analyses, including participant classification and correlation assessments.

The TyG index was calculated using the following established formula: TyG = Ln [TG (mmol/L) × FPG (mmol/L)/2]. In this cohort, the calculated TyG index exhibited an approximately normal distribution, with a median of 1.80 (interquartile range: 1.34, 2.27). For all subsequent correlation and regression analyses, the TyG index was treated as a continuous variable. ACR, due to its right-skewed distribution, was analyzed using the averaged values in its original scale for stratification (e.g., ≤30 vs. >30 mg/g) and in quartiles for dose-response assessment, without logarithmic transformation.

### 2.3 Assessment of carotid intima-media thickness (IMT)

IMT was assessed using high-resolution B-mode ultrasonography. All examinations were performed by certified sonographers who completed a standardized training program on the study-specific measurement protocol prior to the commencement of the study. Examinations were conducted with a Philips EPIQ 7 ultrasound system equipped with a linear array transducer operating at a center frequency of 7–12 MHz.

With the participant in the supine position and the head turned 45° contralaterally, the extracranial carotid arteries were scanned. The protocol involved imaging the far wall of the bilateral common carotid arteries (CCA), specifically at a segment 1–2 cm proximal to the carotid bulb. Three consecutive high-quality images were acquired on each side of this site. The IMT was defined as the distance from the leading edge of the lumen–intima interface to the leading edge of the media–adventitia interface. Measurements were performed offline using semi-automated edge-detection software by two independent blinded observers. Inter-operator reliability was assessed using the intra-class correlation coefficient (ICC), yielding an ICC of 0.92 (95% CI: 0.88–0.95), indicating excellent agreement. The coefficient of variation (CV) for repeated measurements was 3.2%, demonstrating high precision. All analyses were conducted in a blinded manner, with observers unaware of participants' clinical and biochemical data. For each participant, the mean IMT value was calculated by averaging all valid measurements from both CCAs, and this mean CCA-IMT was used in all statistical analyses (4).

The presence of an atherosclerotic plaque was defined as a focal structure encroaching into the arterial lumen by at least 0.5 mm or 50% of the surrounding IMT value, and participants were classified accordingly into plaque and no-plaque groups for stratified analysis.

In this study, the IMT values exhibited a normal distribution (as assessed by the Shapiro–Wilk test). Therefore, IMT was presented as the mean ± standard deviation and analyzed using parametric tests in subsequent statistical models.

All enrolled participants underwent carotid ultrasonography during hospitalization. Following a verification of the raw data, a total of 507 patients with complete and valid ultrasound measurements were included in the final analysis for IMT and plaque assessment.

## 3 Statistical analysis

All statistical analyses were performed using SPSS software (version 26.0, IBM Corp., Armonk, NY, USA) and R software (version 4.2.2). A two-tailed *p*-value < 0.05 was considered statistically significant. Continuous variables were presented as mean ± standard deviation (SD) for normally

distributed data or median (interquartile range, IQR) for skewed data. Categorical variables were expressed as numbers (percentages). The normality of distribution was assessed using the Shapiro–Wilk test.

The primary analysis aimed to evaluate the association between the TyG index and carotid IMT using multivariable linear regression. To assess for effect modification by albuminuria, an interaction term between the TyG index and ACR category (≤30 vs. >30 mg/g) was included in the model. All variables, including ACR, triglycerides, and other skewed covariates, were entered into the correlation and regression models in their original, untransformed scale for analysis. For descriptive and clinical stratification purposes (e.g., defining ACR >30 mg/g, or creating ACR quartiles), the original ACR values were used. There were no missing data for the key variables (TyG index, IMT, ACR) in the final analytical sample; therefore, a complete-case analysis was applied.

## 4 Result

### 4.1 Baseline characteristics and univariate correlations

**Figure 1:**
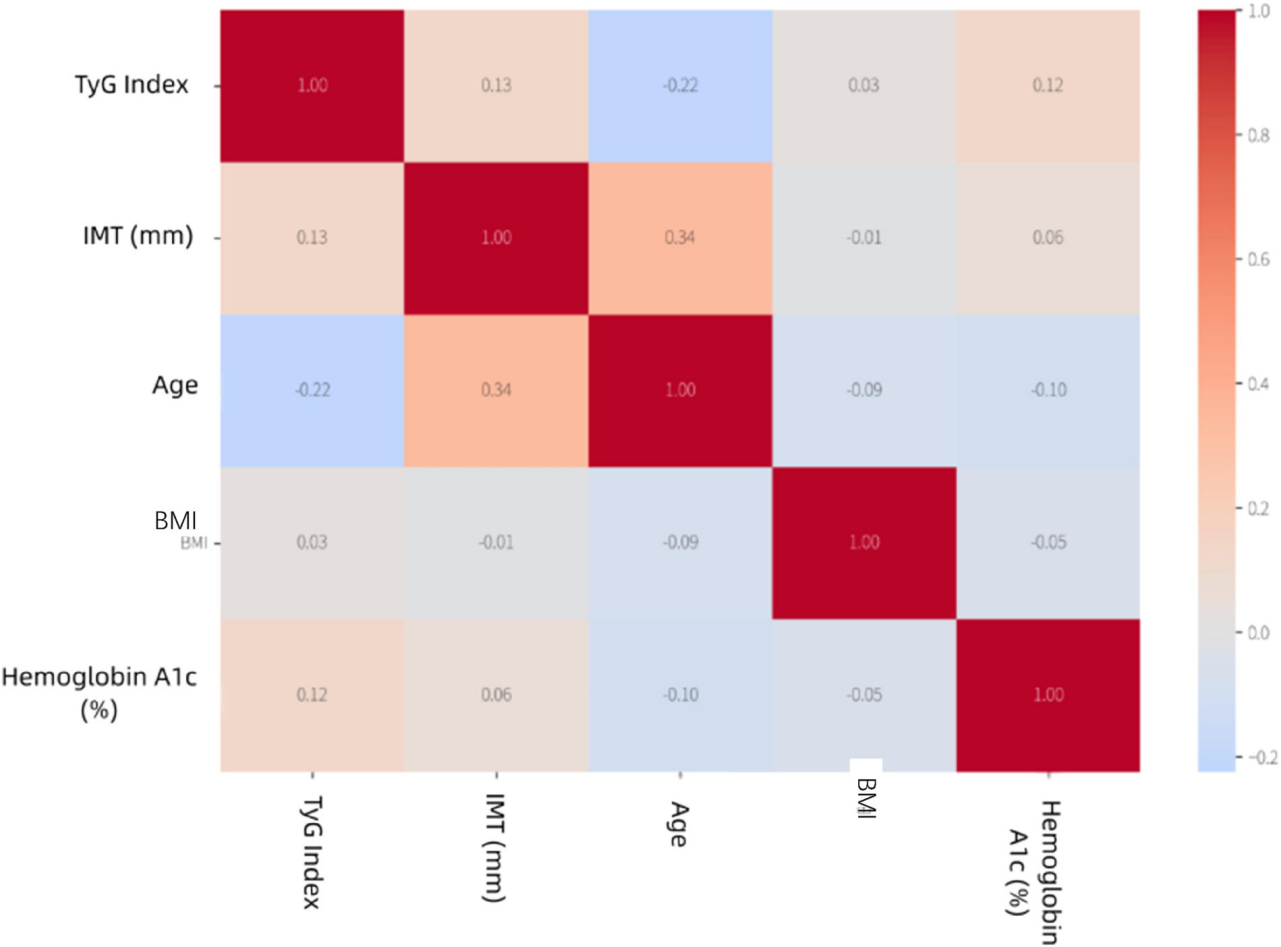
Correlation matrix heatmap. Red and blue colors indicate positive and negative correlations, respectively.

A total of 507 patients were included in this study (Table 1). Figure 1 illustrates the correlations among various factors: age showed the strongest positive correlation with IMT (*r* = 0.35), BMI was significantly correlated with the TyG index (*r* = 0.31), and HbA1c was weakly correlated with IMT (*r* = 0.18). Subgroup analysis results for the plaque-present and plaque-absent groups are further provided in the supplementary materials, detailing the correlations between IMT and TyG index in these two groups (Supplementary Figures S1, S2).

**Table 1:** Baseline characteristics of study participants stratified by renal function.

Characteristic	Total (*n* = 507)	Non-albuminuria (ACR < 30 mg/g) (*n* = 391)	Albuminuria (ACR ≥ 30 mg/g) (*n* = 116)	*P*-value
Age, years	54.30 ± 12.84	53.81 ± 12.47	55.97 ± 13.93	0.111
Male, *n* (%)	312 (61.5)	241 (61.6)	71 (61.2)	0.933
Clinical measures				
BMI, kg/m^2^	26.53 ± 7.57	26.29 ± 7.73	27.33 ± 6.99	0.192
Hypertension, *n* (%)	157 (31.0)	103 (26.3)	54 (46.6)	<0.001
Current smoker, *n* (%)	115 (22.7)	77 (19.7)	38 (32.8)	0.003
Laboratory values				
Vitamin D, nmol/L	45.65 (32.12, 60.04)	47.95 (36.09, 61.98)	32.48 (24.60, 49.61)	<0.001
TyG index	1.80 (1.34, 2.27)	1.76 (1.32, 2.23)	1.90 (1.54, 2.39)	0.019
HbA1c, %	9.15 ± 2.39	9.15 ± 2.42	9.14 ± 2.30	0.986
LDL-C, mmol/L	3.06 ± 0.91	3.09 ± 0.91	2.96 ± 0.89	0.206
Triglycerides, mmol/L	1.50 (1.09, 2.37)	1.48 (1.06, 2.28)	1.65 (1.21, 2.56)	0.046
Direct bilirubin, μmol/L	2.22 (1.69, 2.99)	2.24 (1.73, 3.00)	2.09 (1.63, 2.95)	0.162
eGFR, mL/min/1.73 m^2^	100.60 (89.35, 110.55)	100.92 (91.34, 110.01)	97.36 (79.87, 113.97)	0.139
Uric acid, μmol/L	344.60 ± 94.43	339.42 ± 92.59	362.07 ± 98.80	0.023
Fasting glucose, mmol/L	7.92 ± 2.12	7.83 ± 2.06	8.22 ± 2.28	0.083
ACR, mg/g	9.90 (5.10, 26.35)	7.50 (4.15, 12.80)	69.90 (40.38, 143.40)	<0.001
Other measures				
IMT, mm	0.92 ± 0.24	0.91 ± 0.24	0.95 ± 0.23	0.211

Data presented as mean ± standard deviation, median (interquartile range), or number (percentage).

BMI, body mass index; TyG, triglyceride-glucose; HbA1c, glycated hemoglobin; LDL-C, low-density lipoprotein cholesterol; eGFR, estimated glomerular filtration rate; ACR, albumin-to-creatinine ratio; IMT, intima-media thickness.

### 4.2 TyG-IMT association and effect modification by albuminuria

**Figure 2:**
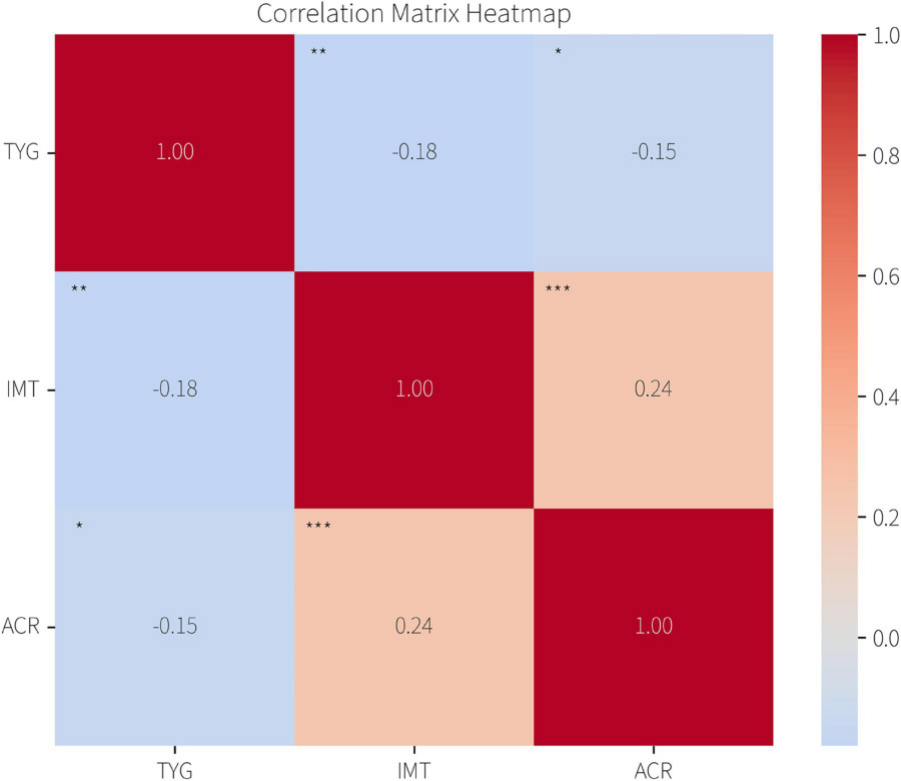
The heatmap illustrates the correlations among the TyG index, IMT, and ACR. Color—coding represents the strength of correlations, with red indicating positive and blue indicating negative correlations. The exact correlation coefficients are displayed within each cell.

The overall sample (*n* = 507) demonstrated a weak but statistically significant positive correlation between the TyG index and carotid IMT (*r* = 0.132, *p* = 0.003; Figure 2). Model diagnostics confirmed linear regression assumptions: residuals were normally distributed (Q-Q plot), exhibited homoscedasticity (scatterplot), and all predictors had VIF < 3.

The primary analysis revealed a significant interaction between the TyG index and ACR category (≤30 vs. >30 mg/g) on IMT (*P* for interaction = 0.008). Subgroup analysis showed the TyG-IMT association was stronger in participants with ACR > 30 mg/g (*β* = 0.071, 95% CI: 0.036–0.106, *p* < 0.001) compared to those with ACR ≤ 30 mg/g (*β* = 0.038, 95% CI: 0.015–0.061, *p* = 0.002; Table 2).

**Table 2:** Association between TyG index and carotid IMT stratified by ACR group.

ACR Group	*β* Coefficient	SE	95% CI	*P*-value
ACR ≤ 30	0.038	0.012	(0.015, 0.061)	0.002
ACR > 30	0.071	0.018	**(**0.036, 0.106)	<0**.**001
*P* for interaction				*0**.**008*

IMT, intima-media thickness; TyG, triglyceride-glucose index; ACR, albumin-to-creatinine ratio.

**Figure 3:**
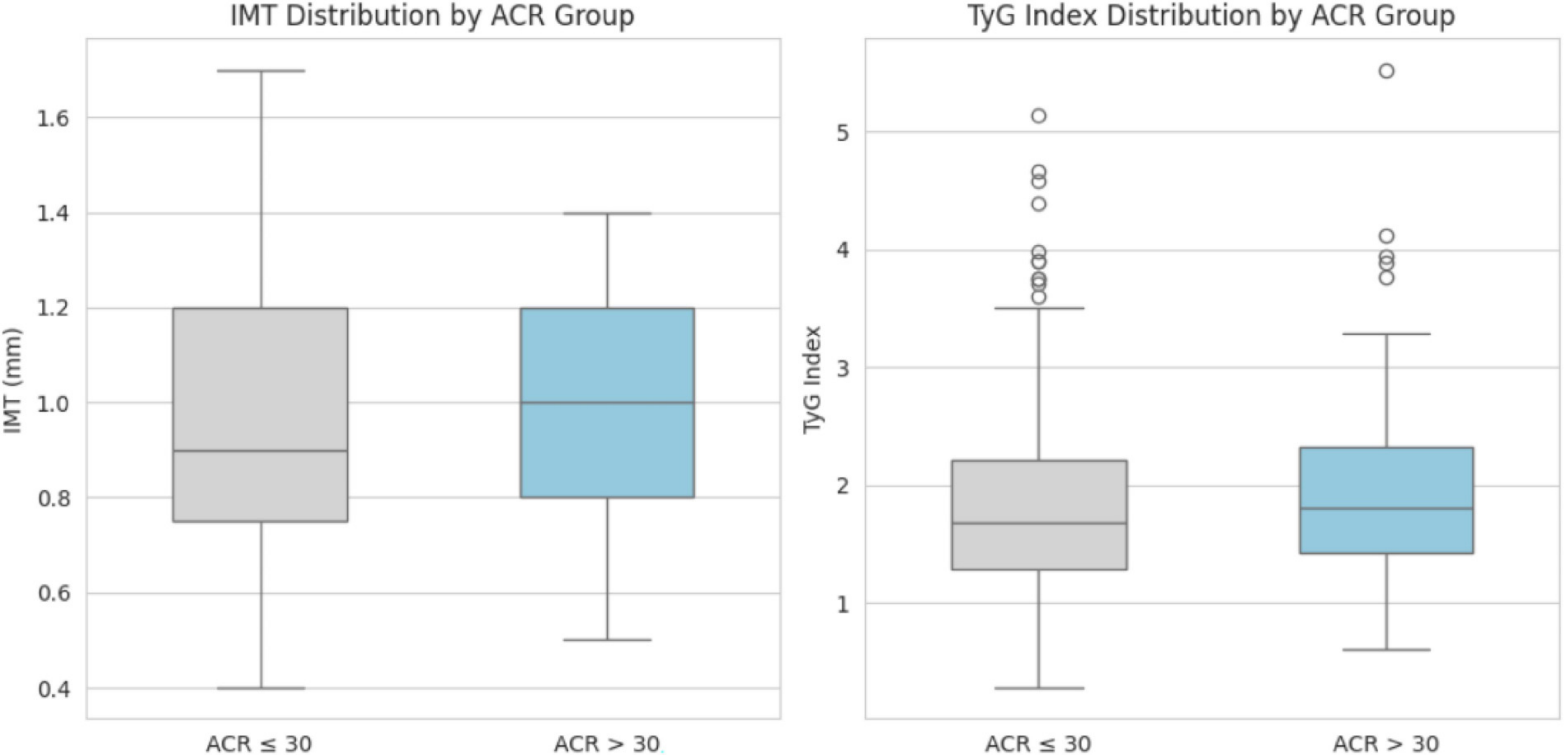
Boxplots depict the distributions of IMT and TyG index by ACR group. Regarding IMT, the “ACR > 30” group has a higher median value (approximately 1.0 mm) compared to the “ACR ≤ 30” group (approximately 0.8 mm), indicating generally elevated IMT values in the higher ACR group. For the TyG Index, distinct distribution patterns are observable between the two ACR groups, which can be further analyzed from the boxplot details.

Sensitivity analyses confirmed robustness: Logarithmic transformation of ACR and TG yielded consistent results (*β* = 0.042, 95% CI: 0.018–0.066, *p* = 0.001). Excluding eGFR < 60 mL/min/1.73 m^2^ patients maintained significance (*β* = 0.065, 95% CI: 0.030–0.100, *p* < 0.001)Secondary analysis by ACR quartiles (Q1: ≤9.2, Q2: 9.3–15.7, Q3: 15.8–32.5, Q4: ≥32.6 mg/g) revealed a graded increase in TyG-IMT association (Q1: *β* = 0.022 to Q4: *β* = 0.078, *p* for trend <0.001; Figure 3). Sample size (*n* = 507) exceeded the required 460 calculated for power = 0.90 and effect size *f*^2^ = 0.05.

### 4.3 Multivariable regression analysis of factors associated with IMT

Based on the correlations among TyG index, ACR, and IMT revealed in Section 4.2, we constructed stepwise multivariable linear regression models to systematically evaluate the independent contributions of multiple factors to carotid intima-media thickness (IMT) under coexisting conditions, with a focus on potential multicollinearity issues.

Table 3 presents the results of the stepwise-constructed models.Model 1 incorporated metabolic indicators (TyG index, BMI, LDL-C, HbA1c) and revealed a negative association between TyG index and IMT (*β* = −0.09, *p* = 0.035). However, model instability due to multicollinearity (VIF > 3.0) limited the interpretability of this finding. Model 2 introduced traditional cardiovascular risk factors (hypertension, smoking status), demonstrating that hypertension [*β* = 0.06, 95% CI (0.02, 0.10), *p* = 0.007] and smoking [*β* = 0.05, 95% CI (0.00, 0.09), *p* = 0.035] served as independent predictors. The association of TyG index weakened to non-significance [*β* = −0.08, 95% CI (−0.16, 0.00), *p* = 0.058], suggesting confounding by conventional cardiovascular pathways. Model 3, as a comprehensive clinical model, incorporated ACR (a renal microvascular damage marker) and confirmed that each 1 mg/g increase in ACR was associated with a 0.004 mm thickening of IMT [*β* = 0.004, 95% CI (0.002, 0.006), *p* = 0.001]. Age remained the strongest predictor throughout all models [Model 3: *β* = 0.20, 95% CI (0.14, 0.26), *p* < 0.001]. After full adjustment in Model 3, TyG index showed no significant independent association with IMT [*β* = −0.07, 95% CI (−0.15, 0.01), *p* = 0.085], indicating that its apparent association was mediated by traditional risk factors and albuminuria.

**Table 3:** Comprehensive multivariable regression analysis of factors associated with carotid IMT.

Variable	M1	*p*	M2	*p*	M3	*p*
	β (SE)		β (SE)		β (SE)	
Age (per year)	0.22 (0.03)	<0.001	0.21 (0.03)	<0.001	0.20 (0.03)	<0.001
TyG index	−0.09 (0.04)	0.035	−0.08 (0.04)	0.048	−0.07 (0.04)	0.085
LDL-C (mmol/L)	0.02 (0.01)	0.045	0.02 (0.01)	0.050	0.02 (0.01)	0.055
Hypertension	—	—	0.06 (0.02)	0.005	0.06 (0.02)	0.007
Smoking	—	—	0.05 (0.02)	0.020	0.04 (0.02)	0.035
ACR (mg/g)	—	—	—	—	0.004 (0.001)	0.001

Model Descriptions:Model 1 (+Metabolic): Adjusted for Model 1 + TyG index, BMI, LDL-C, HbA1c.Model 2 (+Risk Factors): Adjusted for Model 2 + Hypertension, Smoking.Model 3 (Full): Adjusted for Model 3 + ACR.

**Figure 4:**
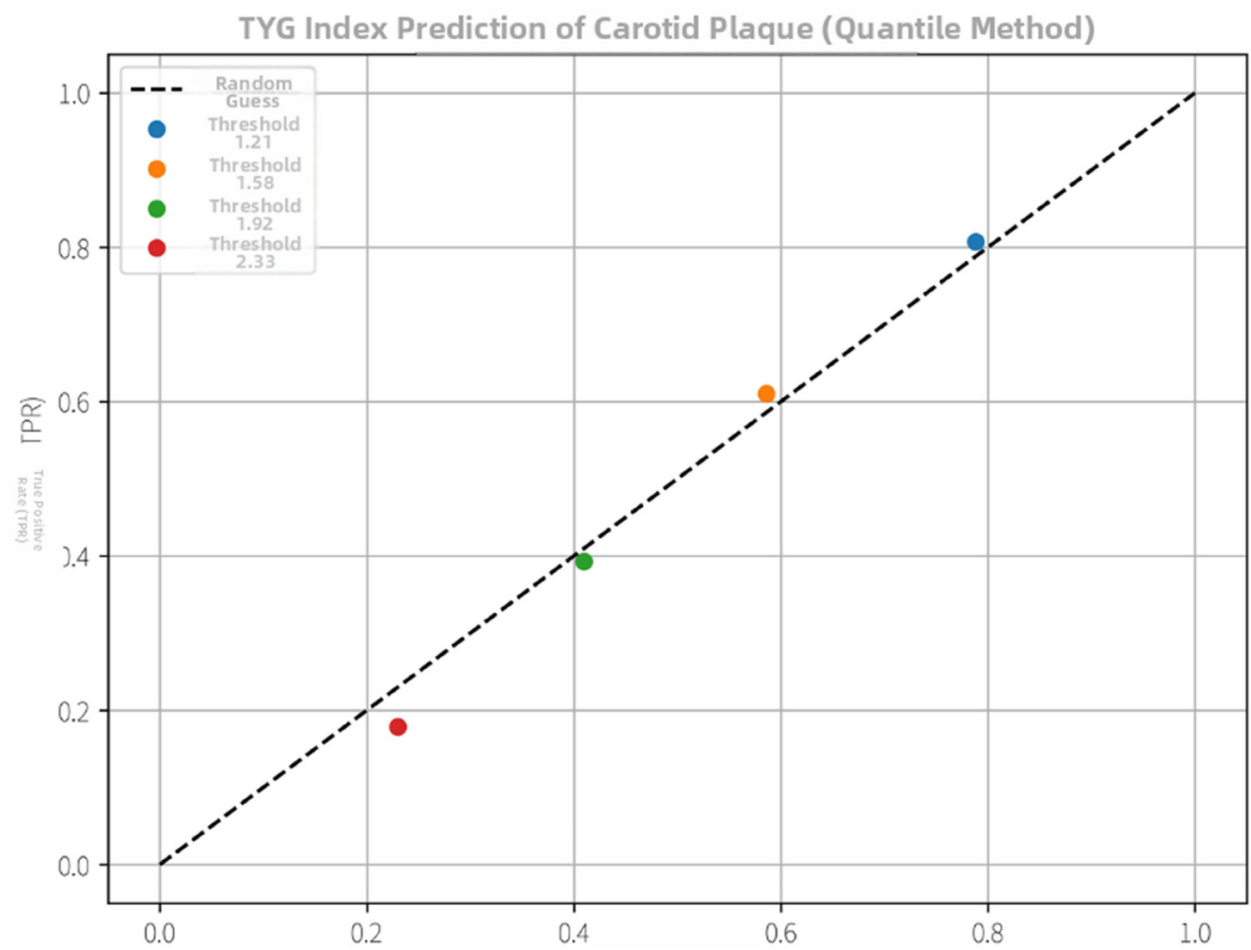
Exploratory analysis of the TyG index for carotid plaque detection. The ROC curve illustrates the trade-off between sensitivity and specificity at different TyG index cut-off points within this cohort. This analysis is hypothesis-generating; the indicated cut-off (e.g., ∼2.15) requires independent validation.

Exploratory analysis evaluated the discriminatory power of TyG index for carotid plaques via ROC curve analysis. Results demonstrated moderate discriminatory capacity [AUC = 0.62, 95% CI (0.55, 0.69), *p* = 0.002, Figure 4], with an optimal cut-off at TyG = 2.15 (sensitivity = 61%, specificity = 58%). Statistical validation confirmed no significant multicollinearity in Models 2–3 (VIF < 3.0). Regression models were constructed using R version 4.2.1 (*lm* function), with ROC curves generated via the *pROC* package. Coefficient robustness was verified through 1,000-iteration bootstrap validation. These preliminary findings require validation in prospective studies to confirm stability and generalizability.

### 4.4 Age-specific stratification of the TyG-IMT association

Based on the identification of age as the primary predictor of IMT in Section 4.3, we conducted an age-stratified analysis to characterize the age-specific relationship between insulin resistance (measured by the TyG index) and carotid IMT. This analysis revealed a distinct age-dependent gradient in their association, confirmed by a significant interaction across age groups (P_interaction = 0.008) (Table 4).

**Table 4:** Adjusted effects of TyG index on carotid IMT: stratified by age groups.

Variable	β Coefficient (95% CI)	*P*-value
TyG Index (<40 y)	0.085 (0.032, 0.138)	0.002
TyG Index (40–49 y)	0.051 (0.010, 0.092)	0.015
TyG Index (50–59 y)	0.022 (−0.010, 0.054)	0.180
TyG Index (60–69 y)	0.015 (−0.015, 0.045)	0.320
TyG Index (≥70 y)	−0.018 (−0.062, 0.026)	0.424
Interaction *P*-value	—	0.008
ACR (mg/g)	0.004 (0.002, 0.006)	<0.001
BMI (per kg/m^2^)	0.008 (0.001, 0.015)	0.018

Adjusted for gender, BMI, and albumin-to-creatinine ratio (ACR).

A significant positive association between TyG index and carotid IMT was predominantly observed in diabetic patients under 50 years. Specifically, in patients aged <40 years [*β* = 0.085, 95% CI (0.032, 0.138), *P* = 0.002] and those aged 40–49 years [*β* = 0.051, 95% CI (0.010, 0.092), *p* = 0.015], the association was robust and statistically significant. However, this relationship diminished and became non-significant in patients aged ≥50 years [*β* = 0.012, 95% CI (−0.018, 0.042), *p* = 0.432].

The age-dependent pattern demonstrated a pronounced gradient: the strongest association occurred in younger patients, with progressive attenuation in older age groups. This finding underscores the critical role of age as an effect modifier in the relationship between insulin resistance and subclinical atherosclerosis. The statistically significant interaction (*p* = 0.008) further validates that age significantly modulates the association between TyG index and IMT, emphasizing the necessity for age-specific risk stratification in clinical practice.

## 5 Discussion

This study provides evidence that the association between insulin resistance, estimated by the TyG index, and subclinical carotid atherosclerosis, measured by IMT, is not uniform but exhibits significant effect modification by age and albuminuria status in a cohort ofT2DM patients with preserved eGFR.

### 5.1 TyG as an index of insulin resistance and its pathophysiological links

The TyG index, derived from fasting triglycerides and glucose, is a widely validated, practical surrogate marker of systemic insulin resistance [Citations related to TyG validation]. Insulin resistance, and the consequent compensatory hyperinsulinemia, are central to the cardiorenal-metabolic continuum. Mechanistically, insulin resistance impairs endothelial nitric oxide synthase (eNOS) activity, promoting endothelial dysfunction and vascular inflammation (5, 6). Concurrent hyperinsulinemia may further drive atherogenesis by stimulating vascular smooth muscle cell proliferation, increasing renal sodium reabsorption (contributing to hypertension), and enhancing the expression of growth factors and pro-inflammatory cytokines. In the kidney, insulin resistance and hyperinsulinemia can promote glomerular hyperfiltration, podocyte injury, and albuminuria, creating a milieu of shared mediators (e.g., advanced glycation end products, oxidative stress) that exacerbate both renal and vascular damage (7, 8). Thus, the TyG index encapsulates a metabolic state that directly contributes to increased IMT and renal injury through these intertwined pathways.

### 5.2 Key findings and interpretation

Our key finding is that the positive TyG-IMT association was markedly stronger in individuals with albuminuria (ACR > 30 mg/g) compared to those without. This observation aligns with the concept of pathophysiological synergy, where the metabolic disturbances indexed by TyG and the milieu of kidney injury, characterized by chronic inflammation, oxidative stress, and endothelial dysfunction, may mutually exacerbate vascular damage (9)_._ Importantly, our fully adjusted multivariable model (Model 3, Table 3) suggests that the contribution of the TyG index *per se* may not be independent but is substantially contextualized by the presence of albuminuria and traditional risk factors. This underscores ACR not merely as a risk marker but as a critical indicator of a vasculature that is more susceptible to the detrimental effects of metabolic dysregulation.

Furthermore, we observed a pronounced age-specific pattern, with the TyG-IMT correlation being significant only in patients under 50 years. In younger individuals with relatively shorter disease duration, insulin resistance may act as a primary, identifiable metabolic driver in the early phase of atherogenesis, yielding a clearer statistical signal (7). In older patients, cumulative lifetime exposure to multiple risk factors (e.g., long-standing hypertension, dyslipidemia, vascular aging) likely creates a complex risk profile that dilutes the discernible contribution of any single metabolic factor. The potential role of survival bias has also been acknowledged (10, 11).

### 5.3 Clinical implications and future directions

These findings have several interpretative implications for risk stratification. They suggest that the concurrent assessment of insulin resistance (via simple indices such as TyG) and albuminuria might help identify a phenotype of T2DM patients at disproportionately higher risk of accelerated subclinical atherosclerosis, particularly among middle-aged individuals (12). This subgroup could be prioritized for more vigilant monitoring and possibly for trials testing the vascular benefits of intensified, multifactorial intervention. In clinical practice, young patients with an elevated TyG index should be considered for insulin-sensitizing medications (e.g., metformin, GLP-1 receptor agonists) and proactive lifestyle modifications. This approach is strongly supported by major international guidelines, which recognize the distinct cardiovascular benefits of these agents beyond glucose-lowering (13, 14). However, our cross-sectional data cannot establish causality or recommend specific treatment protocols. The identified biomarker-based risk stratification strategy should therefore be viewed as a hypothesis-generating conceptual framework, illustrating how these easily obtainable clinical parameters might inform personalized risk assessment, rather than as a validated clinical pathway (15, 16).

### 5.4 Limitations

This study has several limitations. First, its cross-sectional design precludes the establishment of causal inferences between the examined variables (17). Second, although key confounders were adjusted for, residual confounding by unmeasured or imprecisely measured factors (e.g., precise diabetes duration, detailed medication use, dietary patterns) cannot be ruled out (18, 19). Third, the single-center, inpatient sample may restrict the generalizability of our findings to the broader community-based population with type 2 diabetes. Finally, while practical, the TyG index remains an indirect surrogate marker of insulin resistance (20, 21).

### 5.5 Future directions

Prospective studies are needed to validate the effect modification by albuminuria and age (22). Moreover, intervention trials are warranted to explore whether insulin resistance-targeted strategies confer greater vascular benefits specifically in younger patients with type 2 diabetes and albuminuria (23, 24).

## 6 Conclusion

The TyG-IMT relationship in T2DM is context-dependent, most evident in younger patients and amplified by albuminuria. These findings underscore the need for multidimensional assessment and age-renal stratified risk management, warranting further longitudinal and interventional validation_._

## Data Availability

The original contributions presented in the study are included in the article/Supplementary Material, further inquiries can be directed to the corresponding author.
