# Supplementary material for: Association of the triglyceride-glucose index with carotid intima-media thickness in type 2 diabetes: effect modification by age and albuminuria—a retrospective cross-sectional study
Source: Front Cardiovasc Med. 2026 Mar 4;13:1711633. doi: 10.3389/fcvm.2026.1711633 (PMC12995680; doi:10.3389/fcvm.2026.1711633)
Supplement: Supplementary file 1 [file Datasheet1.docx]

**
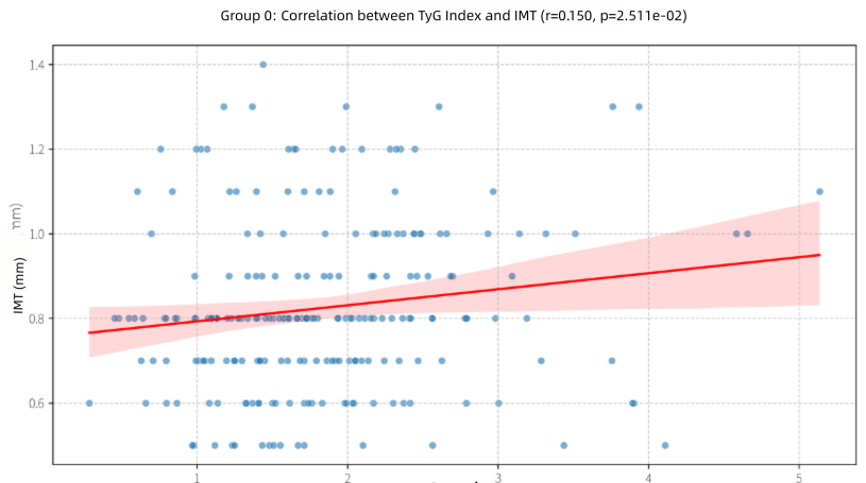

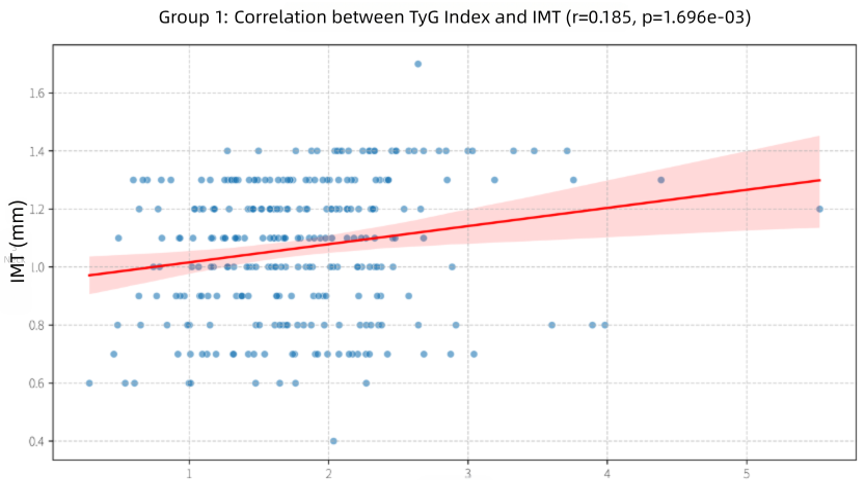
**

**Figure S1 (Group 0: No-plaque group)** shows a positive correlation between the TyG index and IMT, with a correlation coefficient of r=0.150*r*=0.150 (p=2.511×10−2*p*=2.511×10−2; p<0.05*p*<0.05). This statistically significant result suggests that even in individuals without carotid plaques, a higher TyG index is mildly associated with increased IMT.

**Figure S2 (Group 1: Plaque group)** also demonstrates a positive correlation, with a stronger correlation coefficient of r=0.185*r*=0.185 (p=1.696×10−3*p*=1.696×10−3; p<0.01*p*<0.01). This correlation is not only stronger than in the no-plaque group but also highly statistically significant, indicating a more pronounced association between the TyG index and IMT in individuals with existing carotid plaques.

In summary, the TyG index showed a positive correlation with IMT regardless of plaque status. This correlation was stronger in the plaque group, suggesting that the TyG index may hold clinical significance in the assessment of atherosclerosis progression.
